# Evolution of Acidic Mammalian Chitinase Gene (*CHIA*) is Related to Insectivory Feeding in Rodents

**DOI:** 10.1002/ece3.74062

**Published:** 2026-07-22

**Authors:** Yuntao Tian, Zhenyu Xiong, Chengyao Yang, Chaoyang Luo, Xionghui Xu, Yangsong Wu, Qiurong Li, Zhenglang Zhang, Xinya Ma, Jialu Lin, Yuan Mu

**Affiliations:** ^1^ Institute of Eastern‐Himalaya Biodiversity Research Dali University Dali Yunnan China; ^2^ Center for Interdisciplinary Sciences Dali University Dali Yunnan China; ^3^ School of Life Sciences Yunnan Normal University Kunming Yunnan China

**Keywords:** adaptive molecular evolution, *CHIA* gene, insectivory feeding, purifying selection, rodents

## Abstract

Insect exoskeletons rich in chitin form a key dietary component for numerous rodents, yet the molecular evolutionary mechanisms of chitin digestion mediated by the acidic mammalian chitinase gene *CHIA* remain poorly resolved in this hyper‐diverse mammalian order. We systematically analyzed *CHIA* sequences from 37 rodent species spanning distinct insect/chitin intake levels to link gene evolution with insectivorous adaptation. Obligate herbivorous rodents with negligible dietary chitin frequently exhibited *CHIA* deletion or pseudogenization; pseudogenes in *Microtus ochrogaster* underwent significantly relaxed selection, while pseudogenized *Rhynchomys soricoides* retained stable selective constraint, possibly due to occasional invertebrate feeding and residual physiological functions of *CHIA*. Genome‐wide site tests identified five robust positively selected residues concentrated in the catalytic and disulfide bond domains critical for chitinase activity. The universal one‐ratio model revealed pervasive purifying selection across all rodents (*ω* = 0.258), whereas free‐ratio, branch‐site and aBSREL models detected lineage‐specific episodic positive selection in omnivorous taxa with sustained chitin consumption including *Rattus rattus* . Multi‐ratio branch models further confirmed divergent evolutionary constraints: lineages ingesting chitin bore stronger purifying selection than zero‐chitin feeders, with selection intensity correlated to dietary invertebrate proportion. This study provides the first comprehensive rodent‐wide evidence that dietary insectivory drives adaptive divergence of *CHIA*, uncovering distinct molecular degeneration and selection patterns shaped by varying chitin intake and illuminating the genomic basis of mammalian dietary adaptation.

## Graphical abstract

This study provides the first systematic investigation into the molecular evolution of the acidic mammalian chitinase (CHIA) gene across 37 rodent species with diverse diets. Results demonstrate that the CHIA gene has been lost or pseudogenized in obligate herbivores, while being maintained under purifying selection in chitin‐consuming lineages, with selection strength positively correlating with dietary chitin content. The identification of positively selected sites within critical functional domains offers novel insights into the genetic mechanisms driving dietary adaptation to insectivory in mammals.

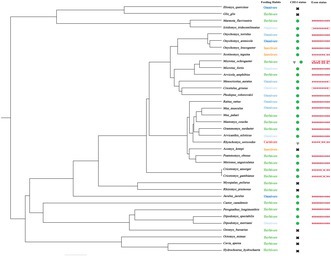

## 1 Introduction

Chitin, a linear β‐1, 4‐linked polymer of N‐acetyl‐D‐glucosamine (GlcNAc), is the second most abundant polysaccharide in nature after cellulose. It occurs predominantly in the exoskeletons of arthropods, the largest animal phylum, which includes Crustacea and Insecta, and in fungal cell walls, where it serves as an important energy source for many vertebrates (Aam et al. 2010; Hamid et al. 2013; Van Huis 2016). Efficient extraction of energy from chitin represents a highly economical metabolic pathway. The digestion and hydrolysis of dietary chitin primarily depend on chitinases, glycoside hydrolases (GHs) that catalyze the biodegradation of β‐1,4‐glycosidic bonds in aminopolysaccharides (Chen et al. 2020). Acidic mammalian chitinase (AMCase), encoded by the *CHIA* gene, belongs to glycoside hydrolase family18 (GH18) and is widely expressed and relatively conserved across mammals (Hussain and Wilson 2013).

Mammals are a highly diverse group distributed across different habitats worldwide. Insects are consumed by numerous lineages, including Primates and Chiroptera, among others. Insect predation is considered an adaptive pressure driving the evolution of morphological traits in mammals (Cartmill 1992, 2012). For example, in insectivorous primates, adaptations such as molar teeth with crests for masticating insect exoskeletons (Kay 1975), simple gastrointestinal tracts with a low ratio of stomach to small intestine (Chivers and Hladik 1980), smaller body size (Kay 1984), and grasping hands (Bishop 1964) have been proposed. At the molecular level, digestion of insect exoskeletons in mammals primarily relies on GH18 family members, particularly AMCase encoded by the *CHIA* gene (Bussink et al. 2007; Singh et al. 2021). Studies indicate that *CHIA* often exhibits higher enzymatic activity in insectivorous mammals, such as insect‐eating mice and pangolins (*Manis* spp.) (Whitaker Jr et al. 2004; Strobel et al. 2013; Ma et al. 2018). Molecular analyses further reveal that the *CHIA* gene has undergone adaptive evolution in various insectivorous mammalian lineages. For instance, within Chiroptera, many insectivorous and omnivorous bat species possess 2 or 3 functional gene copies (Wang et al. 2020). In primates, species with high dietary insect content, such as the *Tarsius syrichta* and *Otolemur garnettii*, which exhibit strong purifying selection on the *CHIA* gene (Janiak et al. 2018). Conversely, in several obligate carnivore lineages, the *CHIA* gene has become pseudogenized (Tabata et al. 2022). Despite these findings, research on the adaptive evolution of the *CHIA* gene in mammals has largely focused on Chiroptera, Primates, and Carnivora, with limited systematic investigation in Rodentia, one of the largest mammalian orders.

As the most speciose order of mammals, rodents are distributed globally, and their dietary diversity has played a crucial role in their adaptive radiation. Rodent diets are commonly classified into herbivory, omnivory, and carnivory, with insectivory recognized as a distinct subset; however, insects are also consumed by omnivorous and carnivorous species (Verde Arregoitia and D'Elía 2021). Research on insectivorous rodents has primarily emphasized morphological, physiological, biochemical, and ecological adaptations. Morphologically, such rodents exhibit distinct snout, dental, and skull features compared to those with other diets, including more elongated snouts, relatively upright and smaller teeth, and reduced temporal fossae (Samuels 2009; Alhajeri and Steppan 2018; Missagia et al. 2021). Physiologically and biochemically, omnivorous rodents such as mice display elevated expression levels of acidic chitinase relative to herbivores and carnivores (Tabata et al. 2018; Chu et al. 2025). At the genetic level, prior studies have shown that the *CHIA* gene has been lost or pseudogenized in certain non‐insectivorous rodent lineages, resulting in loss of function (Emerling et al. 2018). Given the extensive species richness, broad dietary variation, and widespread inclusion of insects in rodent diets, investigating the adaptive evolutionary mechanisms of the *CHIA* gene in this group is highly significant.

Currently, research on insectivory in rodents remains largely confined to macroscopic analyses, and the molecular basis, particularly the evolutionary patterns of the acidic mammalian chitinase (*CHIA*) gene across the entire order, is still poorly understood. Therefore, this study examines whether insectivory in rodents has driven adaptive evolution of the *CHIA* gene, aiming to establish a theoretical foundation for understanding the underlying molecular mechanisms and to elucidate broader adaptive evolutionary processes associated with insectivory in rodents and mammals generally. This work also contributes to the general understanding of adaptive evolution in organisms.

## 2 Materials and Methods

### 2.1 Data Collection and Processing

This work used publicly available data. All information is available at the NCBI (https://www.ncbi.nlm.nih.gov/) database and no ethics statement was required in such occasions. In order to cover as comprehensively as possible the species and their dietary information, 37 rodent species that have a range of different dietary ecologies were selected, which covered 12 families and 30 genera, insect consumption varies from 0% (e.g., *Psammomys obesus* , which are eating leaves or fruit) to 100% (e.g., *Scotinomys teguina* , most of mainly feeding on invertebrate), making them an ideal group for a comparative study of dietary adaptations associated with insectivory. Generally, this dataset includes three major dietary types, namely, Herbivorous, Omnivorous and Carnivorous. In order to better investigate whether there are differences in the selection pressure on genes among different dietary groups, we further classified them based on the content of chitin in the food. The dietary information and composition of each species were obtained from published research (Faurby et al. 2018), the proportion of invertebrates in diet was adopted as a proxy for dietary chitin intake. Species were classified into three trophic categories: (1) lineages with high chitin intake: invertebrate proportion > 60%; (2) lineages with low chitin intake: 10% ≤ invertebrate proportion ≤ 60%; (3) lineages with low chitin intake: invertebrate proportion < 10% (Emerling et al. 2018) (Table S1).

The coding sequences (CDS) of most species were obtained from the NCBI (Table S1), used the CDS of a representative placental mammal with a well‐annotated genome ( *Mus musculus* ) as the query, with approximately 50 bp flanking sequences retained on both sides of each exon, then through the TBtools‐II v2.326 genome BLAST to obtain the sequences of some species (Chen et al. 2023) (Table S2). The *CHIA* sequences were aligned based on their amino acid translations using online PRANK (https://www.ebi.ac.uk/goldman‐srv/webprank/), and then deleted the gaps and non‐homologous regions by using GBLOCK; then we corrected the multiple sequences alignment (MSA) in MEGA 7 (Kumar et al. 2016) by eye.

### 2.2 Identification of Inactivating Mutations

The intact *CHIA* sequences of *Mus musculus* were used to screen for inactivating mutations, including mutations in initiation codons, frame shift insertions and deletions, premature stop codons, and mutations at the GT/AG splice sites of intron exon boundaries. All candidate inactivating mutations were verified via BLAST against the whole‐genome sequences of corresponding taxa retrieved from NCBI.

To further identify the decline in enzymatic activity induced by mutations in functional sites and domains, we aligned functional site sequences of all species with published mammalian reference sequences. Previous studies have demonstrated that amino acid substitutions at sites 214 and 216 in *Canis lupus* (Carnivora) and site 128 in *Bos taurus* (Artiodactyla) reduce chitinase catalytic activity (Tabata et al. 2022, 2023). Furthermore, we compared the conserved catalytic motif DXXDXDXE specific to active chitinases across all available taxa and analyzed six conserved cysteine residues responsible for chitin binding (Tjoelker et al. 2000; Olland et al. 2009).

### 2.3 Selection Pressure Analysis

Selection pressure analysis is mainly based on the ratio *ω* of nonsynonymous (*d*_*N*_) to synonymous substitution (*d*_*S*_)of coding sequences, which is used as the main basis for detecting natural selection: *ω* > 1, = 1, or < 1 indicates that the gene is subjected to positive, neutral, or purifying selection, respectively. The *ω* value was evaluated using the CodeML program in the PAML 4.9 package (Yang 2007). The likelihood ratio test statistic (2ΔL), which approximates a chi‐square (*χ* ^2^) distribution, was used to compare nested likelihood models. Positively selected signals were identified using BEB analysis with posterior probabilities (PPs) of ≥ 0.8 (Yang et al. 2005). We used the TimeTree database (http://www.timetree.org/) (Kumar et al. 2017) to obtain the Rodentia phylogenetic tree for subsequent analysis (Figure S1), with *Homo sapiens* designated as the outgroup for subsequent analysis. These methodologies collectively enhance the accuracy of the analysis, thereby facilitating a more precise understanding and interpretation of the role of natural selection in the adaptive evolution of species.

We performed site modeling (M8 and M8a) using the CodeML program in the PAML package to detect sites under positive selection (Yang 2007). Additionally, the codon model FUBAR (Fast, Unconstrained Bayesian Approximation for Inferring Selection) was used to estimate codons under positive selection; the sites with a posterior probability higher than 0.9 were all considered as being under selection (Murrell et al. 2013). Sites detected by at least two methods were regarded as robust candidates for positive selection.

To investigate the evolutionary rate across the entire rodent lineage, we utilized the one‐ratio (M0) model implemented in the CodeML program of the PMAL 4.9 package (Yang 2007). The M0 model posits that all species within the dataset are subjected to the same selective pressure, which implies a uniform *ω* value across all branches and nodes of the phylogenetic tree. Subsequently, we compared the M0 model with the free ratio model (M1) to determine whether evolutionary lineages exhibit varying evolutionary rates. The free ratio model allows for the assumption that each branch possesses an independent *ω* value, which was also implemented via the CodeML program in PAML 4.9 (Yang 2007). To determine whether sites subject to positive selection are restricted to specific evolutionary lineages, the more rigorous branch‐site model was employed using the CodeML program from PAML 4.9 (Yang 2007). This model permits each site on a designated branch to possess its own *ω* value, initially categorizing the phylogeny into foreground branches (those of primary interest) and background branches. The alternative hypothesis model Ma (positive selection model: 0 < *ω*_0_ < 1, *ω*_1_ > 1, and *ω*_2_ ≥ 1) is compared to the null hypothesis model Ma0 (neutral model: 0 < *ω*_0_ < 1, *ω*_1_ = 1, and *ω*_2_ = 1). In this investigation, each terminal branch was treated as a foreground branch. When the pairwise comparison of models (Ma vs. Ma0) shows significant differences and *ω* > 1, the positive selection model Ma is accepted. Additionally, sites with a posterior probability (PP) > 0.8 are considered to be under positive selection. The significance of all pairwise models was determined through LRTs based on Chi‐square tests, with a significant level at *p* < 0.05 (Yang 2007).

Given that non‐independent mutations at adjacent sites (e.g., dinucleotide substitutions) may cause false positives, we further adopted the BS + MNM (Branch‐Site test incorporating multinucleotide mutation) model to reverify identified selection signals and eliminate bias induced by correlated neighboring substitutions (Kosakovsky Pond et al. 2020). To avoid subjectivity from manual foreground branch definition and improve robustness, the aBSREL (adaptive Branch‐Site Random Effects Likelihood) model implemented in HyPhy v2.5.33 was additionally used (Smith et al. 2015). Under exploratory mode, all branches across the phylogeny were screened for positive selection without prior dietary grouping (Venkat et al. 2018).

To better understand the selective pressure, a series of evolutionary models were compared in the likelihood. We first used the M0 model (Model A), which assumed that all branches in the phylogenetic tree have a common value, and compared it with the null hypothesis (Model B), which assumed that the common value in the phylogenetic tree is 1. To further understand whether the selective pressure on the lineages leading to pseudogenes was relaxed, we constructed Model C, which assumed that the branches with pseudogene had their own selection pressure *ω*_2_, while the background branches without pseudogenization had *ω*_1_, and then compared Model C with Model A. To further confirm whether the selective pressure on the lineages leading to pseudogenes was completely relaxed, we built Model D, which assumed that the branches with pseudogene had their own selection pressure *ω*_2_ = 1, while the selective pressure of background branches was *ω*_1_, and then compared Model C with Model D.

To verify relaxed selection, we ran RELAX (HyPhy v2.5.33) (Wertheim et al. 2015). The two pseudogenized lineages were specified as foreground branches and all other lineages as background. The parameter *k* was interpreted as: *K* < 1: relaxed purifying selection, *K* = 1: unchanged selection, *K* > 1: intensified selection. LRT with *p* < 0.05 was used for significance testing.

To understand whether the adaptive evolution of *CHIA* gene occurs in different branches, branch models were employed using the “two‐ratio (2ω),” and “three‐ratio (3ω),” models, which were implemented in CodeML (Yang 1998). First, to test whether different selective pressures act on the lineages with no chitin intake and lineages with chitin intake rodent lineages, the one‐ratio model that enforces the same *ω* ratio for all lineages was compared with the 2ω model that allows one *ω* ratio for all branches and another for all remaining branches (lineages with no chitin intake species) (Figure S2A). Second, to explore the rate variation between lineages with low chitin intake and lineages with high chitin intake species, we used the 3ω model, which assumes independent *ω* values for lineages with low chitin intake and lineages with high chitin intake species and the remaining lineages (Figure S2B).

### 2.4 Protein 3D Structure Analysis

To gain an insight into the functional implications of the detected positively selected sites, we mapped these sites to the three‐dimensional (3D) structures of each related protein downloaded from the AlphaFold website (https://alphafold.com/) using PyMOL (DeLano 2002). Functional information about the sites and domains of each candidate protein was obtained from the UniProt website (http://www.uniprot.org/) (UniProt Consortium 2019) and InterPro (https://www.ebi.ac.uk/interpro/) (Paysan‐Lafosse et al. 2023).

## 3 Results

### 3.1 Sequence Characteristics of the *CHIA* Gene and Diet

**FIGURE 1:**
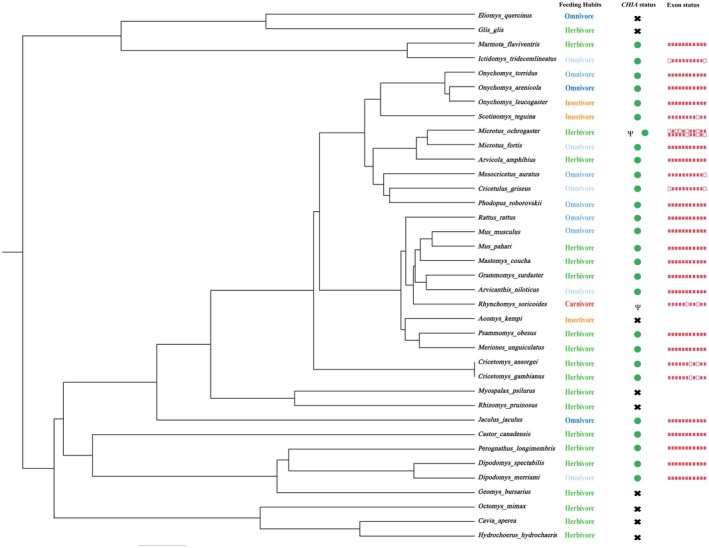
The Characteristics of *CHIA* gene and feeding information in rodents (The left is phylogenetic relationship of rodents. Pseudogenes are indicated by the symbol “Ψ”. For omnivorous species, the color gradient is used to indicate dietary preference: The lighter shade indicates more herbivorous, the darker shade indicates more carnivorous. Green circle indicates the main partial sequences are used for analysis. Cross indicates the sequences are not obtained or extremely short. The rightmost red box represents the exon status: filled indicates the presence of an exon, while empty indicates the absence).

A total of 37 *CHIA* gene sequences were used in this study. Inactivating mutations, such as premature stop codons and splice site mutations, were detected in lineages with no chitin intake. For example, premature stop codons were identified in the *CHIA* gene of *R. soricoides* and *M. ochrogaster* . Moreover, the *CHIA* gene sequence cannot be identified in some genomes of herbivorous species, for example, *Cavia aperea* and *Octomys mimax* , and partial *CHIA* gene fragments were found in some other herbivorous species, for example, *Eliomys quercinus* , *Myospalax psilurus* , and *Rhizomys pruinosus* . Notably, two copies of the *CHIA* gene were identified in *M. ochrogaster* , one of which was pseudogenized due to a premature stop codon. Generally, the *CHIA* gene remained intact and a single copy in most species (Figure 1).

### 3.2 Characterization of *CHIA* Sequence

**FIGURE 2:**
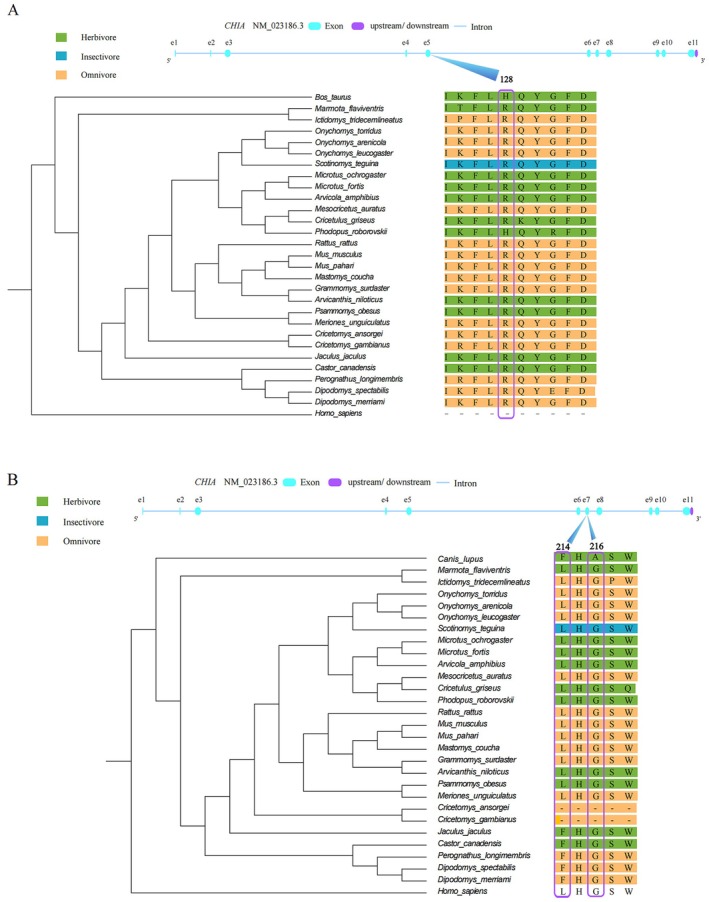
(A) Sequence alignment of the 128th amino acid site in the coding region of the *CHIA* gene. (B) Sequence alignment of the 214th and 216th amino acid site in the coding region of the *CHIA* gene.

**FIGURE 3:**
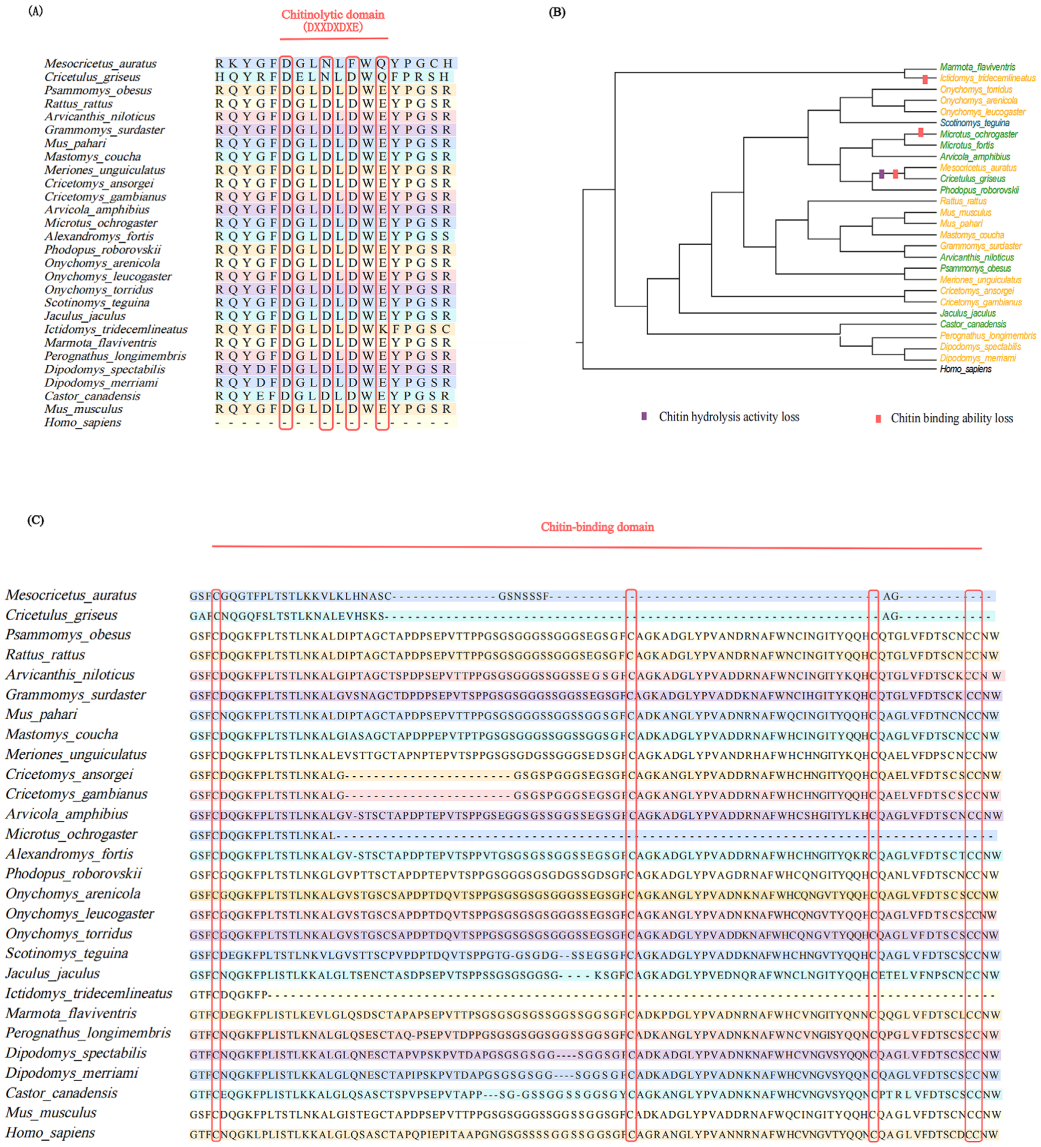
Comparison of predicted ancestral protein sequences of the nine mammalian chitinase paralogs. (A) Conserved amino acid residues of the canonical chitinolytic domain active site (DXXDXDXE). (B) Summary of the evolution of chitinase paralogs functionality. (C) Conserved cysteine residues of the chitin‐binding domain.

To compare the functional potential of chitinase encoded by *CHIA*, we characterized *CHIA* evolutionary patterns in rodents using functionally critical active‐site residues from *B. taurus* and *C. lupus familiaris* as references. *Phodopus roborovskii* harbors identical amino acid substitutions documented in *B. taurus* *CHIA* (Figure 2A). *Jaculus jaculus* , *Castor canadensis* , *Perognathus longimembris* , *Dipodomys spectabilis* , and *Dipodomys merriami* share the same substitution at site 214 observed in *C. lupus* *CHIA*, whereas no mutation was detected at site 216 (Figure 2B). We further examined the catalytic motif (DXXDXDXE) for chitin hydrolysis and six conserved cysteine residues responsible for substrate binding. Mutations within the catalytic domain were identified in *Mesocricetus auratus* (three altered sites) and *Cricetulus griseus* (two altered sites). The last four conserved cysteines were completely deleted in *M. auratus* , *C. griseus* , *M. ochrogaster* , and *Ictidomys tridecemlineatus* , while all six cysteines remained intact in the remaining examined sequences (Figure 3).

### 3.3 Selective Pressure Analysis

#### 3.3.1 Positively Selected Sites

**FIGURE 4:**
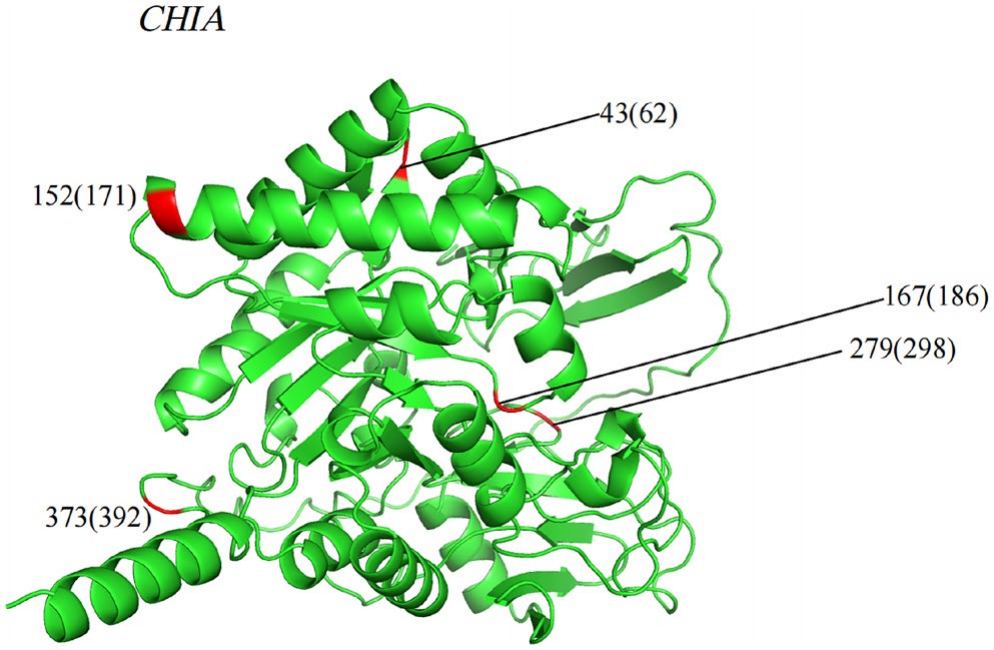
Annotation of the positively selected sites of the *CHIA* gene on the 3D protein structure. The first number is for the order of analysis sequences used in this study. The numbers in brackets are for the true order of query sequences.

To investigate the presence of positively selected sites across the entire lineage, two methods of site model (M8 vs. M8a in PAML and FUBBR in datamonkey) were employed. Results from M8 and M8a did not detect any positively selected sites. However, FUBAR identified a total of 5 positively selected sites in the dataset (Table 1). In order to better understand the potential function of these positively selected sites, we further mapped these sites onto the protein 3D structure (Figure 4). Based on the information from UniProt, all 5 sites were found to be located within the catalytic domain and disulfide bond regions.

**TABLE 1:** Results of the FUBAR model detection.

Gene	Site	*α*	*β*	Prob [*α* > *β*]	Prob [*α* < *β*]	BF [*α* < *β*]	*β*‐*α*
*CHIA*	43	0.632	2.843	0.018	0.947	45.896	2.212
	152	0.502	1.857	0.049	0.918	28.613	1.355
	167	0.396	1.408	0.050	0.920	29.391	1.011
	279	0.382	2.235	0.013	0.974	97.704	1.853
	373	0.464	3.359	0.001	0.991	282.587	2.896

#### 3.3.2 Selective Pressure Among Functional Lineages

The one‐ratio model (M0) analysis of *CHIA* gene across the entire rodent lineage yielded a purifying selection (*ω* = 0.258). Likelihood ratio tests comparing the free‐ratio model (M1) against the one‐ratio model (M0) showed that the M1 model fit significantly better than the M0 model (*p* < 0.05) (Table 2), indicating significant variation in selective pressure among the entire lineage during the *CHIA* gene evolution. Notably, *R. rattus* exhibited positive selection (*ω* = 2.618). Furthermore, we conducted further analyses of the positive selection signals detected on the terminal branches of *I. tridecemlineatus* and *M. auratus* using the branch‐site model, and a total of 19 positively selected sites were identified. Subsequent analyses using the branch‐site mutational nucleotide model (BS + MNM) showed that neither the *I. tridecemlineatus* nor the *M. auratus* clades reached the significance threshold, whereas significant positive selection signals were detected in *R. rattus* (Table 3). In addition, exploratory analysis across the entire tree using the adaptive Branch‐Site Random Effects Likelihood (aBSREL) model detected significant positive selection on the branches of *I. tridecemlineatus* and *R. rattus* , with nine positively selected sites jointly identified by the two methods (Table 3).

**TABLE 2:** Results of the free‐ratio model detection.

Gene	Model	‐lnL	Model comparison	2∆lnL	df	*p*
*CHIA*	M0 (one‐ratio)	10,206.23413	M0 vs. M1	86.785	53	0.002
	M1 (free‐ratio)	10,162.85491				

**TABLE 3:** Results of the branch‐site model detection.

Gene	The branch of	Model	‐lnL	2∆lnL	df	*p*	aBSREL (*p*)	Branch‐site test—multinucleotide mutations (*p*)	Positive site (PP > 80%)
*CHIA*	*Ictidomys tridecemlineatus*	Ma	10,003.366	15.2	1	< 0.01	< 0.05	NS	122 Y 0.825 157 P 0.806 179 S 0.824 180 Q 0.974* 181 L 0.972* 191 M 0.812 198 S 0.810 202 Y 0.995** 205 E 0.995** 213 P 0.994** 218 I 0.952* 220 A 0.983* **282 W 0.915** 283 A 0.833 289 T 0.988* 348 F 0.996**
		Ma0	10,010.960						
	*Mesocricetus auratus*	Ma	10,020.626	5.794	1	0.016	NS	NS	96 G 0.815 119 F 0.906 225 K 0.844
		Ma0	10,017.729						
	*Rattus ruttus*	Ma	10,028.498	0	1	1	< 0.05	< 0.05	**46 N**
		Ma0	10,028.498						

*Note:* Loci marked in bold were jointly detected by at least two methods. *Posterior probability (PP) ≥ 0.95, **PP ≥ 0.99.

#### 3.3.3 Evolutionary Analysis of *CHIA* Pseudogenes

To detect whether selective pressure was relaxed in lineages with pseudogenized *CHIA*, Model C analysis was performed on these branches. Results indicated that the *ω* value on the pseudogenized terminal branch of *M. ochrogaster* was significantly higher than the *ω* value of background branches (Table 4), with *ω*_1_ = 0.244 and *ω*_2_ = 3.174. Comparing Model D with Model C showed no significant difference. In contrast, the pseudogenized terminal branch of *R. soricoides* showed no significant difference in selective pressure compared to the background branches (*ω*_1_ = 0.249, *ω*_2_ = 0.346, *p* > 0.05) (Table 4).

**TABLE 4:** Likelihood and *ω* values estimated under two ratio branch model on pseudogenized branches.

Relative branches	Models	*ω*	‐ln L	np	Models comparison	2∆lnL	*p*
The terminal branch of *Microtus ochrogaster*	A. All branches have one *ω*	0.250	10,384.131	58			
	B. The termina branch has *ω*_2_, others have *ω*_1_	*ω*_1_ = 0.244 *ω*_2_ = 3.174	10,372.707	59	A vs. B	22.848	< 0.01
	C. The termina branch has *ω*_2_ = 1, others have *ω*_1_	*ω*_1_ = 0.244 *ω*_2_ = 1.000	10,374.505	58	B vs. C	3.596	0.058
The terminal branch of *Rhynchomys soricoides*	A. All branches have one *ω*	0.250	10,421.367	58			
	B. The termina branch has *ω*_2_, others have *ω*_1_	*ω*_1_ = 0.249 *ω*_2_ = 0.346	10,420.879	59	A vs. B	0.976	0.323

Further analysis of selection pressure strength using the RELAX model revealed that the selection pressure on *M. ochrogaster1* was significantly relaxed (*K* < 1, *p* < 0.001), while no significant relaxation of selection pressure was detected in *R. soricoides* (*p* = 0.38) (Table 5).

**TABLE 5:** Estimated selection intensity parameter *K*and significance for pseudogene branches based on the RELAX model.

Gene	The branch of	Model	*p*	*K*
*CHIA*	*Microtus ochrogaster1*	RELAX	< 0.001	< 1
	*Rhynchomys soricoides*		0.38	0.76

#### 3.3.4 Selective Patterns of Different Lineages With Chitin Intake

Multi‐ratio models revealed a gradient of selective pressure correlated with chitin intake. The overall *ω* was 0.228 (purifying selection). The result of the two‐ratio model showed that lineages with chitin intake exhibited stronger purifying selection (*ω* = 0.232) than lineages with no chitin intake (*ω* = 0.248). The three‐ratio model revealed that lineages with low chitin intake exhibited the strongest selective constraint (*ω* = 0.227). The comparison with the two‐ratio model showed no statistically significant difference between the models (*p* = 0.438). The *ω* values for each taxon were 0.508, 0.247, and 0.227, respectively (Table 6).

**TABLE 6:** *ω* values estimated under different branch models according to degree of chitin intake *Rodentia*.

Model	‐lnL	np	LRT	Comparison	*p*	*ω* value		
						Lineages with no chitin intake	Lineages with low chitin intake	Lineages with high chitin intake
1ω	10,162.855	109				0.228	0.228	0.228
2ω	10,206.097	57	86.484	2ω vs. 1ω	< 0.01	0.248	0.232	0.232
3ω	10,205.878	58	0.438	3ω vs. 2ω	0.508	0.247	0.227	0.292

## 4 Discussion

Mammals are thought to have descended from small insectivores, and the *CHIA* gene evolved lineage‐specific traits during dietary diversification (Kemp 2005; Emerling et al. 2018). As the most species‐rich mammalian clade with highly heterogeneous diets, rodents play a pivotal role in elucidating the adaptation to feeding habits and the evolutionary pattern of the *CHIA* gene in mammals. As the key gene responsible for chitin digestion, *CHIA* is highly expressed in the stomach of insectivorous and omnivorous animals (Ohno et al. 2016; Uehara et al. 2018, 2021; Tabata et al. 2019). On the contrary, it is not expressed in many herbivorous mammals or functional activity has significantly decreased (Allio et al. 2025; Okawa et al. 2016). Accordingly, the *CHIA* gene may have exhibited a diversified selection model. In this study, the M0 model (*ω* = 0.258) indicated that most functional genes are typically subject to strong purifying selection to preserve essential biological functions (Weinreich et al. 2005; Wollenberg Valero 2020). And then, based on the M1 model, the *CHIA* gene exhibited significant differences, suggesting the *CHIA* gene has undergone divergent evolutionary rates due to facing diverse feeding environments.

Many previous studies have shown that the related genes have been functionally lost or become pseudogenized. For example, in obligate carnivores such as felids and cetaceans, the sweet taste receptor gene *TAS1R2* has been universally inactivated due to loss of sweetness in their foods (Jiang et al. 2012). Whilst, the umami taste receptor gene *TAS1R1* of giant pandas and red pandas has also lost its function because of dietary shift from carnivorous to bamboo‐eating (Hu et al. 2017). In this study, the *CHIA* gene has been extremely short or completely absent in most herbivorous species (e.g., *C. aperea* and *O. mimax* ), and omnivorous *Acomys kempi* and *E. quercinus* , or pseudogenized across many herbivorous rodent species, indecently (Figure 1). Interestingly, we identified a duplication of the *CHIA* gene in *M. ochrogaster* . Among them, one copy is a pseudogene, and another one has lost its vital domain, that is, the chitin‐binding domain. Besides, whether based on the results of PAML or RELAX, the results indicated that the selective pressure has been relaxed in the pseudo‐*CHIA* of *M. ochrogaster*. Our results uncovered that the correlation with no‐chitin intake of this species (Tabata et al. 2022). Furthermore, pseudogenization of *CHIA* was identified in *R. soricoides* , whereas its selective pressure remained unrelaxed, which might be related to its feeding preference for earthworms and small soil invertebrates (Watson 1958). This evolutionary trend is analogous to that observed in walruses ( *Odobenus rosmarus* ), which have no functional *CHIA*s, and 80% of its diet is composed of invertebrates (Emerling et al. 2018). However, the *CHIA* gene did not exhibit any extreme signature such as pseudogenization or deletion. It might be because the pseudogenized *CHIA* retains partial residual functions or participates in immune defense and other biological processes (Wen et al. 2012; Hu et al. 2021). And then the chitinase encoded by the *CHIA* gene has been significantly reduced due to mutations in some key amino acid sites or functional domains, just like dogs and cows (Tabata et al. 2022, 2023). Indeed, some inactive mutations were identified, such as R128H substitution in *P. roborovskii* , which was consistent with the inactivating mutation in *B. taurus* (Tabata et al. 2023), while *J. jaculus* and *D. merriami* carry the L214F substitution, consistent with the inactivating mutation in *C. lupus* (Tabata et al. 2022). All mutated residues mentioned above have been proven to markedly reduce chitinase enzymatic activity (Tabata et al. 2022, 2023).

Generally, the purifying selection is the main adaptive pattern during the species radiation (Janiak et al. 2018), which is consistent with our results. However, the episodic positive selection has been identified among some branches. In the terminal branches of *I. tridecemlineatus* and *M. auratus* , 16 and three positively selected sites were detected, respectively, most of which are located within the catalytic domain and disulfide bond regions critical for chitin degradation, conformational stability, and enzymatic activity (Figure 4) (Kashimura et al. 2015; Chittoor et al. 2020; UniProt Consortium 2019). Notably, site 119 in *M. auratus* lies adjacent to the active center and plays a central role in catalysis and enzyme activity maintenance (Zhang et al. 2002; Cai et al. 2009). Additionally, a single positively selected site (site 46, *ω* = 2.618) was detected in the terminal branch of *R. rattus* . As a generalist omnivore with insects comprising 30% of its diet, it is continuously exposed to dietary chitin, and this positive selection signal may reflect adaptation to chitin intake (Banks and Hughes 2012). Collectively, these findings indicate that in rodents with sustained chitin consumption, *CHIA* maintains strong functional activity through adaptive substitutions at key catalytic sites. The gene copies and positive selection signals detected in herbivorous species, however, may be attributable to factors beyond diet or to limitations in sequence quality, and require further validation.

Rodent species have extensive feeding habits, which have been promoting them much better adaptive radiation. Obviously, the food of many species contains, to varying degrees, many types of insects or invertebrates (Table S1). Further research has found that the *CHIA* gene in some rodent lineages exhibits varying degrees of degeneration. In this study, the 2ω model revealed a significant difference in evolutionary rates between lineages with and without chitin intake, which indicates that in the category of whether there is chitin in food, the evolution of *CHIA* has shown significant differences. 3ω model detected no significant difference between the high‐chitin and no‐chitin groups. We need further investigation to confirm the variation, such as increasing the number of species, more methods, etc.

## 5 Conclusion

Based on an evolutionary analysis of 37 rodent species, this study systematically elucidates the molecular evolutionary patterns of the acidic mammalian chitinase (*CHIA*) gene in relation to varying dietary chitin intake. The results indicate that *CHIA* functional status is strongly associated with species' feeding ecology, exhibiting distinct evolutionary trajectories: in lineages with no chitin intake, the gene is either lost or pseudogenized; in contrast, lineages with chitin intake retain the *CHIA* gene under purifying selection, with selection strength positively correlated to dietary chitin content, and significantly stronger purifying selection is observed in species lineages with chitin intake compared to lineages with no chitin intake. Furthermore, 19 positively selected sites were identified in chitinivorous lineages, many localized within critical functional or structural domains of the enzyme. Collectively, these findings demonstrate that the rodent *CHIA* gene has undergone adaptive evolution shaped by dietary habits. These insights not only enhance understanding of the molecular mechanisms underlying dietary adaptation but also establish a theoretical foundation for future investigations into genes involved in chitin digestion.

## Author Contributions

**Yuntao Tian:** data curation (lead), formal analysis (lead), investigation (lead), methodology (lead), resources (lead), software (lead), writing – original draft (lead). **Zhenyu Xiong:** data curation (equal), formal analysis (equal), resources (equal), software (equal). **Chengyao Yang:** data curation (equal), formal analysis (equal), investigation (equal), methodology (equal), resources (equal), software (equal). **Chaoyang Luo:** formal analysis (equal), resources (equal), software (equal). **Xionghui Xu:** formal analysis (equal), resources (equal), software (equal). **Yangsong Wu:** data curation (equal), software (equal). **Qiurong Li:** data curation (equal), resources (equal). **Zhenglang Zhang:** formal analysis (equal), software (equal). **Xinya Ma:** formal analysis (equal), software (equal). **Jialu Lin:** formal analysis (equal), resources (equal). **Yuan Mu:** funding acquisition (lead), project administration (lead), writing – review and editing (lead).

## Funding

This work was supported by the National Natural Science Foundation of China (NSFC) to YM (No. 32360119), and the start‐up projects on high‐level talent introduction of Dali University to YM (No. KY1916101940).

## Conflicts of Interest

The authors declare no conflicts of interest.

## Supporting information


**Figure S1:** The phylogenetic relationship of Rodentia *CHIA* gene used in this study.

**Figure S2:** The model assumptions of the multi‐ratio branch model. A is for two ratio Additional files model and B is for three ratio model.

**Table S1:** The feeding information and accession ID of rodents in this study.

**Table S2:** List of genome accession numbers used in this study.

## Data Availability

The relative data and Figures S1 and S2, Tables S1 and S2 for this article can be found online at: https://doi.org/10.6084/m9.figshare.32928797.

## References

[ece374062-bib-0001] Aam, B. B. , E. B. Heggset , A. L. Norberg , M. Sørlie , K. M. Vårum , and V. G. H. Eijsink . 2010. “Production of Chitooligosaccharides and Their Potential Applications in Medicine.” Marine Drugs 8, no. 5: 1482–1517. 20559485 10.3390/md8051482 PMC2885077

[ece374062-bib-0002] Alhajeri, B. H. , and S. J. Steppan . 2018. “A Phylogenetic Test of Adaptation to Deserts and Aridity in Skull and Dental Morphology Across Rodents.” Journal of Mammalogy 99, no. 5: 1197–1216.

[ece374062-bib-0003] Allio, R. , S. Teullet , D. Lutgen , et al. 2025. “Transcriptomic Data Reveal Divergent Paths of Chitinase Evolution Underlying Dietary Convergence in Anteaters and Pangolins.” Genome Biology and Evolution 17, no. 2: evaf002. 39780438 10.1093/gbe/evaf002 PMC11789784

[ece374062-bib-0004] Banks, P. B. , and N. K. Hughes . 2012. “A Review of the Evidence for Potential Impacts of Black Rats ( *Rattus rattus* ) on Wildlife and Humans in Australia.” Wildlife Research 39, no. 1: 78–88.

[ece374062-bib-0005] Bishop, A. 1964. “Use of the Hand in Lower Primates.” In Evolutionary and Genetic Biology of Primates, 133–225. Elsevier.

[ece374062-bib-0007] Bussink, A. P. , D. Speijer , J. M. F. G. Aerts , and R. G. Boot . 2007. “Evolution of Mammalian Chitinase (−Like) Members of Family 18 Glycosyl Hydrolases.” Genetics 177, no. 2: 959–970. 17720922 10.1534/genetics.107.075846 PMC2034658

[ece374062-bib-0008] Cai, W. , L. Sha , J. Zhou , Z. Huang , and X. Guan . 2009. “Functional Analysis of Active Site Residues of *Bacillus thuringiensis* WB7 Chitinase by Site‐Directed Mutagenesis.” World Journal of Microbiology and Biotechnology 25, no. 12: 2147–2155.

[ece374062-bib-0009] Cartmill, M. 1992. “New Views on Primate Origins.” Evolutionary Anthropology: Issues, News, and Reviews 1, no. 3: 105–111.

[ece374062-bib-0010] Cartmill, M. 2012. “Primate Origins, Human Origins, and the End of Higher Taxa.” Evolutionary Anthropology: Issues, News, and Reviews 21, no. 6: 208–220. 10.1002/evan.21324 23280918

[ece374062-bib-0011] Chen, C. , Y. Wu , J. Li , et al. 2023. “TBtools‐II: A “One for All, All for One” Bioinformatics Platform for Biological Big‐Data Mining.” Molecular Plant 16, no. 11: 1733–1742. 37740491 10.1016/j.molp.2023.09.010

[ece374062-bib-0012] Chen, W. , X. Jiang , and Q. Yang . 2020. “Glycoside Hydrolase Family 18 Chitinases: The Known and the Unknown.” Biotechnology Advances 43: 107553. 32439576 10.1016/j.biotechadv.2020.107553

[ece374062-bib-0013] Chittoor, B. , B. Krishnarjuna , R. A. V. Morales , and R. S. Norton . 2020. “The Single Disulfide‐Directed β‐Hairpin Fold: Role of Disulfide Bond in Folding and Effect of an Additional Disulfide Bond on Stability.” Australian Journal of Chemistry 73, no. 4: 312–320.

[ece374062-bib-0014] Chivers, D. J. , and C. M. Hladik . 1980. “Morphology of the Gastrointestinal Tract in Primates: Comparisons With Other Mammals in Relation to Diet.” Journal of Morphology 166, no. 3: 337–386. 7441763 10.1002/jmor.1051660306

[ece374062-bib-0015] Chu, D. , H. Zhang , Z. Shang , N. Liu , H. Fu , and S. Yuan . 2025. “Gut Microecology of Four Sympatric Desert Rodents Varies by Diet.” Ecology and Evolution 15, no. 3: e70992. 40027415 10.1002/ece3.70992 PMC11868701

[ece374062-bib-0016] DeLano, W. L. 2002. “The PyMOL Molecular Graphics System.” http://www.pymol.org/.

[ece374062-bib-0017] Emerling, C. A. , F. Delsuc , and M. W. Nachman . 2018. “Chitinase Genes (*CHIAs*) Provide Genomic Footprints of a Post‐Cretaceous Dietary Radiation in Placental Mammals.” Science Advances 4, no. 5: eaar6478. 29774238 10.1126/sciadv.aar6478 PMC5955627

[ece374062-bib-0018] Faurby, S. , M. Davis , R. Ø. Pedersen , S. D. Schowanek , A. Antonelli1 , and J. C. Svenning . 2018. “PHYLACINE 1.2: The Phylogenetic Atlas of Mammal Macroecology.” Ecology 99, no. 11: 2626. 29989146 10.1002/ecy.2443

[ece374062-bib-0019] Hamid, R. , M. A. Khan , M. Ahmad , et al. 2013. “Chitinases: An Update.” Journal of Pharmacy & Bioallied Sciences 5, no. 1: 21–29. 23559820 10.4103/0975-7406.106559 PMC3612335

[ece374062-bib-0020] Hu, C. , Z. Ma , J. Zhu , et al. 2021. “Physiological and Pathophysiological Roles of Acidic Mammalian Chitinase (*CHIA*) in Multiple Organs.” Biomedicine & Pharmacotherapy 138, no. 4: 111465. 34311522 10.1016/j.biopha.2021.111465

[ece374062-bib-0021] Hu, Y. , Q. Wu , S. Ma , et al. 2017. “Comparative Genomics Reveals Convergent Evolution Between the Bamboo‐Eating Giant and Red Pandas.” Proceedings of the National Academy of Sciences 114, no. 5: 1081–1086. 10.1073/pnas.1613870114 PMC5293045 28096377

[ece374062-bib-0022] Hussain, M. , and J. B. Wilson . 2013. “New Paralogues and Revised Time Line in the Expansion of the Vertebrate GH18 Family.” Journal of Molecular Evolution 76, no. 4: 240–260. 23558346 10.1007/s00239-013-9553-4

[ece374062-bib-0023] Janiak, M. C. , M. E. Chaney , and A. J. Tosi . 2018. “Evolution of Acidic Mammalian Chitinase Genes (*CHIA*) is Related to Body Mass and Insectivory in Primates.” Molecular Biology and Evolution 35, no. 3: 607–622. 29216399 10.1093/molbev/msx312

[ece374062-bib-0024] Jiang, P. , J. Josue , X. Li , et al. 2012. “Major Taste Loss in Carnivorous Mammals.” Proceedings of the National Academy of Sciences of the United States of America 109, no. 13: 4956–4961. 22411809 10.1073/pnas.1118360109 PMC3324019

[ece374062-bib-0025] Kashimura, A. , M. Kimura , K. Okawa , et al. 2015. “Functional Properties of the Catalytic Domain of Mouse Acidic Mammalian Chitinase Expressed in *Escherichia coli* .” International Journal of Molecular Sciences 16, no. 2: 4028–4042. 25689423 10.3390/ijms16024028 PMC4346942

[ece374062-bib-0026] Kay, R. F. 1984. “On the Use of Anatomical Features to Infer Foraging Behavior in Extinct Primates.” In Adaptations for Foraging in Nonhuman Primates: Contributions to an Organismal Biology of Prosimians, Monkeys, and Apes, 21–53. Columbia University Press.

[ece374062-bib-0027] Kay, R. F. 1975. “The Functional Adaptations of Primate Molar Teeth.” American Journal of Physical Anthropology 43, no. 2: 195–215. 810034 10.1002/ajpa.1330430207

[ece374062-bib-0028] Kemp, T. S. 2005. The Origin and Evolution of Mammals. Oxford University Press.

[ece374062-bib-0029] Kosakovsky Pond, S. L. , A. F. Y. Poon , R. Velazquez , et al. 2020. “HyPhy 2.5—A Customizable Platform for Evolutionary Hypothesis Testing Using Phylogenies.” Molecular Biology and Evolution 37, no. 1: 295–299. 31504749 10.1093/molbev/msz197 PMC8204705

[ece374062-bib-0030] Kumar, S. , G. Stecher , M. Suleski , and S. B. Hedges . 2017. “TimeTree: A Resource for Timelines, Timetrees, and Divergence Times.” Molecular Biology and Evolution 34, no. 7: 1812–1819. 28387841 10.1093/molbev/msx116

[ece374062-bib-0031] Kumar, S. , G. Stecher , and K. Tamura . 2016. “MEGA7: Molecular Evolutionary Genetics Analysis Version 7.0 for Bigger Datasets.” Molecular Biology and Evolution 33, no. 7: 1870–1874. 27004904 10.1093/molbev/msw054 PMC8210823

[ece374062-bib-0032] Ma, J. E. , L. M. Li , H. Y. Jiang , et al. 2018. “Acidic Mammalian Chitinase Gene Is Highly Expressed in the Special Oxyntic Glands of *Manis javanica* .” FEBS Open Bio 8, no. 8: 1247–1255. 10.1002/2211-5463.12461 PMC6070644 30087830

[ece374062-bib-0033] Missagia, R. V. , B. D. Patterson , D. Krentzel , and F. A. Perini . 2021. “Insectivory Leads to Functional Convergence in a Group of Neotropical Rodents.” Journal of Evolutionary Biology 34, no. 2: 391–402. 33617138 10.1111/jeb.13748

[ece374062-bib-0034] Murrell, B. , S. Moola , A. Mabona , et al. 2013. “FUBAR: A Fast, Unconstrained Bayesian Approximation for Inferring Selection.” Molecular Biology and Evolution 30, no. 5: 1196–1205. 23420840 10.1093/molbev/mst030 PMC3670733

[ece374062-bib-0035] Ohno, M. , M. Kimura , H. Miyazaki , et al. 2016. “Acidic Mammalian Chitinase Is a Proteases‐Resistant Glycosidase in Mouse Digestive System.” Scientific Reports 6, no. 1: 37756. 27883045 10.1038/srep37756 PMC5121897

[ece374062-bib-0036] Okawa, K. , M. Ohno , A. Kashimura , et al. 2016. “Loss and Gain of Human Acidic Mammalian Chitinase Activity by Nonsynonymous SNPs.” Molecular Biology and Evolution 33, no. 12: 3183–3193. 27702777 10.1093/molbev/msw198 PMC5100053

[ece374062-bib-0037] Olland, A. M. , J. Strand , E. Presman , et al. 2009. “Triad of Polar Residues Implicated in pH Specificity of Acidic Mammalian Chitinase.” Protein Science 18, no. 3: 569–578. 19241384 10.1002/pro.63 PMC2760363

[ece374062-bib-0038] Paysan‐Lafosse, T. , M. Blum , S. Chuguransky , et al. 2023. “InterPro in 2022.” Nucleic Acids Research 51, no. D1: D418–D427. 36350672 10.1093/nar/gkac993 PMC9825450

[ece374062-bib-0039] Samuels, J. X. 2009. “Cranial Morphology and Dietary Habits of Rodents.” Zoological Journal of the Linnean Society 156, no. 4: 864–888.

[ece374062-bib-0040] Singh, R. V. , K. Sambyal , A. Negi , S. Sonwani , and R. Mahajan . 2021. “Chitinases Production: A Robust Enzyme and Its Industrial Applications.” Biocatalysis and Biotransformation 39, no. 3: 161–189.

[ece374062-bib-0041] Smith, M. D. , J. O. Wertheim , S. Weaver , B. Murrell , K. Scheffler , and S. L. Kosakovsky Pond . 2015. “Less Is More: An Adaptive Branch‐Site Random Effects Model for Efficient Detection of Episodic Diversifying Selection.” Molecular Biology and Evolution 32, no. 5: 1342–1353. 25697341 10.1093/molbev/msv022 PMC4408413

[ece374062-bib-0042] Strobel, S. , A. Roswag , N. I. Becker , T. E. Trenczek , and J. A. Encarnação . 2013. “Insectivorous Bats Digest Chitin in the Stomach Using Acidic Mammalian Chitinase.” Public Library of Science ONE 8, no. 9: e72770. 24019876 10.1371/journal.pone.0072770 PMC3760910

[ece374062-bib-0043] Tabata, E. , A. Itoigawa , T. Koinuma , et al. 2022. “Noninsect‐Based Diet Leads to Structural and Functional Changes of Acidic Chitinase in Carnivora.” Molecular Biology and Evolution 39, no. 1: msab331. 34897517 10.1093/molbev/msab331 PMC8789059

[ece374062-bib-0044] Tabata, E. , A. Kashimura , A. Kikuchi , et al. 2018. “Chitin Digestibility Is Dependent on Feeding Behaviors, Which Determine Acidic Chitinase mRNA Levels in Mammalian and Poultry Stomachs.” Scientific Reports 8, no. 1: 1461. 29362395 10.1038/s41598-018-19940-8 PMC5780506

[ece374062-bib-0045] Tabata, E. , A. Kashimura , M. Uehara , et al. 2019. “High Expression of Acidic Chitinase and Chitin Digestibility in the Stomach of Common Marmoset ( *Callithrix jacchus* ), an Insectivorous Nonhuman Primate.” Scientific Reports 9, no. 1: 159. 30655565 10.1038/s41598-018-36477-y PMC6336882

[ece374062-bib-0046] Tabata, E. , I. Kobayashi , T. Morikawa , A. Kashimura , P. O. Bauer , and F. Oyama . 2023. “Evolutionary Activation of Acidic Chitinase in Herbivores Through the H128R Mutation in Ruminant Livestock.” Iscience 26, no. 8: 107254. 37502259 10.1016/j.isci.2023.107254 PMC10368815

[ece374062-bib-0047] Tjoelker, L. W. , L. Gosting , S. Frey , et al. 2000. “Structural and Functional Definition of the Human Chitinase Chitin‐Binding Domain.” Journal of Biological Chemistry 275, no. 1: 514–520. 10617646 10.1074/jbc.275.1.514

[ece374062-bib-0048] Uehara, M. , E. Tabata , K. Ishii , et al. 2018. “Chitinase mRNA Levels Determined by qPCR in Crab‐Eating Monkey ( *Macaca fascicularis* ) Tissues: Species‐Specific Expression of Acidic Mammalian Chitinase and Chitotriosidase.” Genes 9, no. 5: 244. 29747453 10.3390/genes9050244 PMC5977184

[ece374062-bib-0049] Uehara, M. , E. Tabata , M. Okuda , et al. 2021. “Robust Chitinolytic Activity of Crab‐Eating Monkey ( *Macaca fascicularis* ) Acidic Chitinase Under a Broad pH and Temperature Range.” Scientific Reports 11, no. 1: 15470. 34326426 10.1038/s41598-021-95010-w PMC8322401

[ece374062-bib-0050] UniProt Consortium . 2019. “UniProt: A Worldwide Hub of Protein Knowledge.” Nucleic Acids Research 47, no. D1: D506–D515. 30395287 10.1093/nar/gky1049 PMC6323992

[ece374062-bib-0051] Van Huis, A. 2016. “Edible Insects Are the Future?” Proceedings of the Nutrition Society 75, no. 3: 294–305. 26908196 10.1017/S0029665116000069

[ece374062-bib-0052] Venkat, A. , M. W. Hahn , and J. W. Thornton . 2018. “Multinucleotide Mutations Cause False Inferences of Lineage‐Specific Positive Selection.” Nature Ecology & Evolution 2, no. 8: 1280–1288. 29967485 10.1038/s41559-018-0584-5 PMC6093625

[ece374062-bib-0053] Verde Arregoitia, L. D. , and G. D'Elía . 2021. “Classifying Rodent Diets for Comparative Research.” Mammal Review 51, no. 1: 51–65.

[ece374062-bib-0054] Wang, K. , S. Tian , J. Galindo‐González , L. M. Dávalos , Y. Zhang , and H. Zhao . 2020. “Molecular Adaptation and Convergent Evolution of Frugivory in Old World and Neotropical Fruit Bats.” Molecular Ecology 29, no. 22: 4366–4381. 32633855 10.1111/mec.15542

[ece374062-bib-0055] Watson, M. R. 1958. “The Chemical Composition of Earthworm Cuticle.” Biochemical Journal 68, no. 3: 416–420. 13522639 10.1042/bj0680416 PMC1200371

[ece374062-bib-0056] Weinreich, D. M. , R. A. Watson , and L. Chao . 2005. “Sign Epistasis and Genetic 490 Constraint on Evolutionary Trajectories.” Evolution 59, no. 6: 1165–1174. 16050094

[ece374062-bib-0057] Wen, Y. Z. , L. L. Zheng , L. H. Qu , F. J. Ayala , and Z. R. Lun . 2012. “Pseudogenes Are Not Pseudo Any More.” RNA Biology 9, no. 1: 27–32. 22258143 10.4161/rna.9.1.18277

[ece374062-bib-0058] Wertheim, J. O. , B. Murrell , M. D. Smith , S. L. Kosakovsky Pond , and K. Scheffler . 2015. “RELAX: Detecting Relaxed Selection in a Phylogenetic Framework.” Molecular Biology and Evolution 32, no. 3: 820–832. 25540451 10.1093/molbev/msu400 PMC4327161

[ece374062-bib-0059] Whitaker, J. O., Jr. , H. K. Dannelly , and D. A. Prentice . 2004. “Chitinase in Insectivorous Bats.” Journal of Mammalogy 85, no. 1: 15–18.

[ece374062-bib-0060] Wollenberg Valero, K. C. 2020. “Aligning Functional Network Constraint to Evolutionary Outcomes.” BMC Evolutionary Biology 20, no. 1: 58. 32448114 10.1186/s12862-020-01613-8 PMC7245893

[ece374062-bib-0061] Yang, Z. 1998. “Likelihood Ratio Tests for Detecting Positive Selection and Application to Primate Lysozyme Evolution.” Molecular Biology and Evolution 15, no. 5: 568–573. 9580986 10.1093/oxfordjournals.molbev.a025957

[ece374062-bib-0062] Yang, Z. 2007. “PAML 4: Phylogenetic Analysis by Maximum Likelihood.” Molecular Biology and Evolution 24, no. 8: 1586–1591. 17483113 10.1093/molbev/msm088

[ece374062-bib-0063] Yang, Z. , W. S. W. Wong , and R. Nielsen . 2005. “Bayes Empirical Bayes Inference of Amino Acid Sites Under Positive Selection.” Molecular Biology and Evolution 22, no. 4: 1107–1118. 15689528 10.1093/molbev/msi097

[ece374062-bib-0064] Zhang, H. , X. Huang , T. Fukamizo , S. Muthukrishnan , and K. J. Kramer . 2002. “Site‐Directed Mutagenesis and Functional Analysis of an Active Site Tryptophan of Insect Chitinase.” Insect Biochemistry and Molecular Biology 32, no. 11: 1477–1488. 12530215 10.1016/s0965-1748(02)00068-1

