# Supplementary figures and images for: Evolution of Acidic Mammalian Chitinase Gene (CHIA) is Related to Insectivory Feeding in Rodents
Source: Ecol Evol. 2026 Jul 22;16(7):e74062. doi: 10.1002/ece3.74062 (PMC13391239; doi:10.1002/ece3.74062)

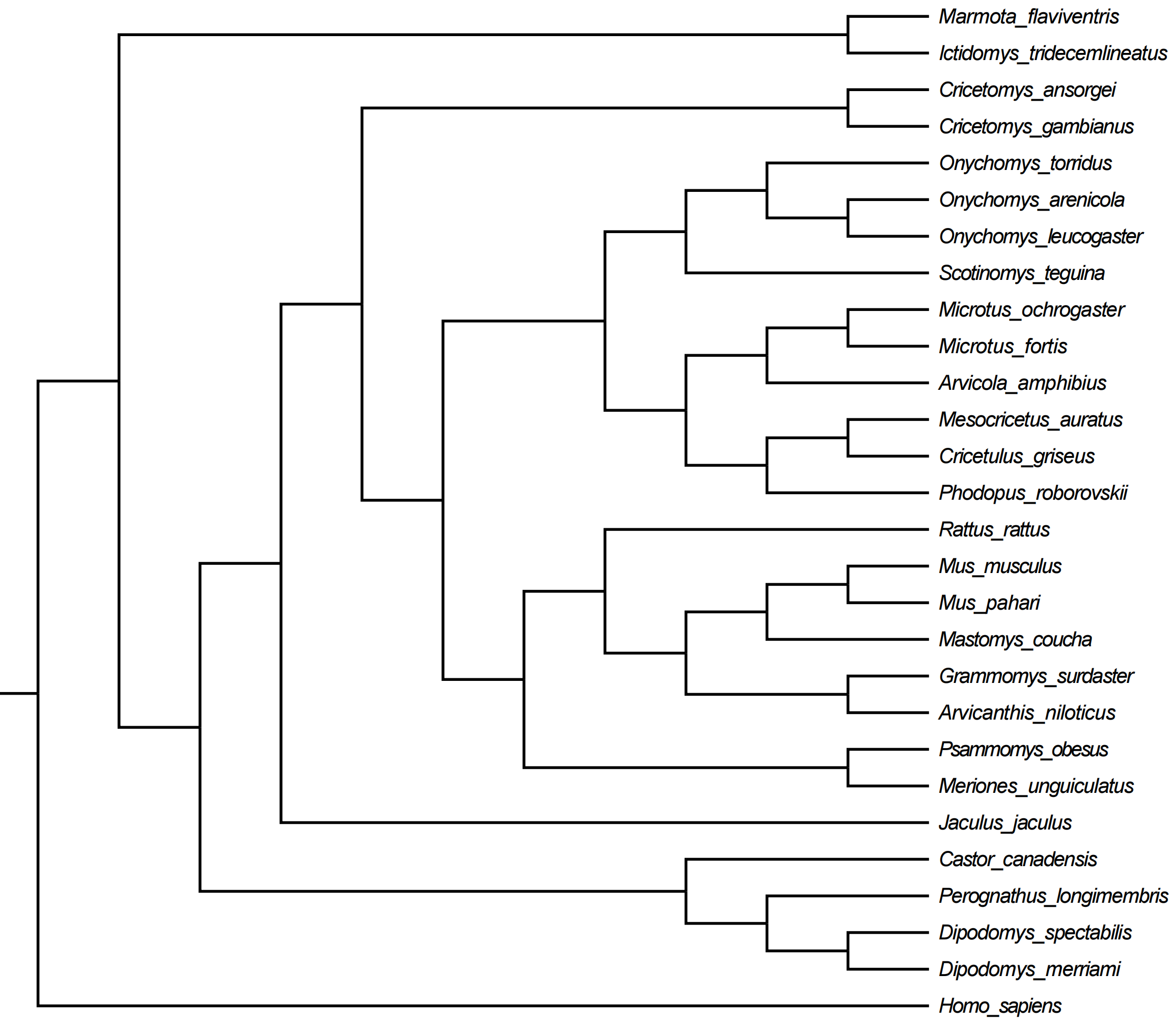

Supplement: Supplementary file 1 — Figure S1: The phylogenetic relationship of Rodentia CHIA gene used in this study. [file ECE3-16-e74062-s003.png]

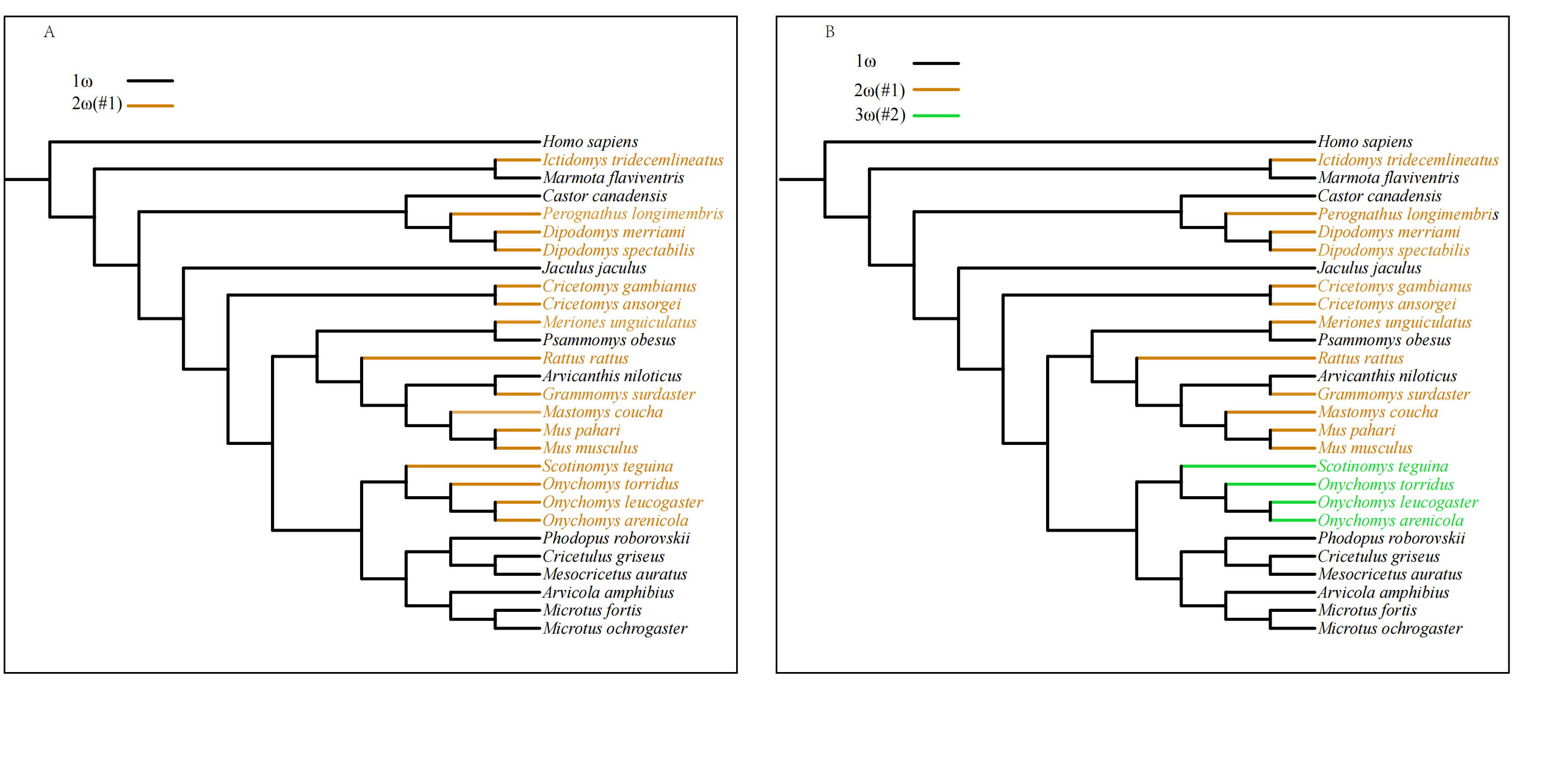

Supplement: Supplementary file 2 — Figure S2: The model assumptions of the multi‐ratio branch model. A is for two ratio Additional files model and B is for three ratio model. [file ECE3-16-e74062-s004.png]
