# Supplementary material for: Evolution of Acidic Mammalian Chitinase Gene (CHIA) is Related to Insectivory Feeding in Rodents
Source: Ecol Evol. 2026 Jul 22;16(7):e74062. doi: 10.1002/ece3.74062 (PMC13391239; doi:10.1002/ece3.74062)
Supplement: Supplementary file 4 — Table S2: List of genome accession numbers used in this study. [file ECE3-16-e74062-s002.docx]

| **Species** | **GenBank** | **Scaffold N50** | **Contig N50** |
| --- | --- | --- | --- |
| *Onychomys arenicola* | GCA_949786405.1 | 108.4 Mb | 26.2 Mb |
| *Onychomys leucogaster* | GCA_949786385.1 | 94.1 Mb | 19.2 Mb |
| *Scotinomys teguina* | GCA_049901635.1 | 1.4 Mb | 1.4 Mb |
| *Cricetomys ansorgei* | GCA_026225945.1 | 12.8 Mb | 12.8 Mb |
| *Cricetomys gambianus* | GCA_004027575.1 | 110 kb | 80.8 kb |
| *Rhynchomys soricoides* | GCA_019843965.1 | 7.9 Mb | 79.8 kb |
| *Myospalax psilurus* | GCA_049190895.1 | 89.5 Mb | 20.1 Mb |
| *Octomys mimax* | GCA_002564305.1 | 4.9 kb | 4.5 kb |
| *Acomys kempi* | GCA_907164505.1 | 125.2 Mb | 33.6 kb |
| *Glis glis* | GCA_004027185.1 | 30.3 kb | 26.1 kb |
| *Eliomys quercinus* | GCA_051143605.1 | 108.1 Mb | 51.7 Mb |
| *Geomys bursarius* | GCA_040207655.1 | 13.1 Mb | 11.8 Mb |
| *Hydrochoerus hydrochaeris* | GCA_004027455.1 | 202.2 kb | 148.5 kb |
| *Cavia aperea* | GCA_000688575.1 | 27.9 Mb | 1 kb |
| *Rhizomys pruinosus* | GCA_009823505.1 | 2.2 Mb | 103.3 kb |

****Table S2****
